# Rectal Cancer Radiotherapy Response Prediction: Retrospective Study of Development of a Deep Learning–Based Radiomics Model

**DOI:** 10.2196/77313

**Published:** 2026-03-17

**Authors:** Yiqun Li, Hengchang Liu, Qiang Wei, Zhenqi Wang, Zhen Hu

**Affiliations:** 1 Department of Colorectal and Anal Surgery, Shanxi Provincial People's Hospital, No. 29 Shuangtasi Street, Taiyuan, 030012, China, 86 0351-4960080; 2 Department of Colorectal Surgery, National Cancer Center/National Clinical Research Center of Cancer/Cancer Hospital, Chinese Academy of Medical Sciences and Peking Union Medical College, Beijing, China

**Keywords:** deep learning, radiomics, rectal cancer, radiotherapy, Transformer model

## Abstract

**Background:** Radiotherapy (RT) is a cornerstone of multimodal treatment for rectal cancer (RC); yet, substantial interindividual variability in treatment response persists. Deep learning (DL)–based radiomics offers potential for pre-RT response prediction to support personalized decision-making.

**Objective:** This study aimed to develop and compare multiple DL radiomics models for predicting RT response in RC, with emphasis on the performance and clinical utility of Transformer architectures.

**Methods:** In this single-center retrospective study, 2000 pathologically confirmed patients with RC who received standard RT were included. Pretreatment computed tomographic and dynamic contrast-enhanced magnetic resonance images and clinical variables were collected. Treatment response was categorized according to RECIST (Response Evaluation Criteria in Solid Tumors) version 1.1 as good (complete or partial response) or poor (stable or progressive disease). The primary analysis used magnetic resonance imaging (MRI)–only input; computed tomography (CT) was used for registration and quality control and evaluated in a late-fusion CT + MRI sensitivity analysis. Data were randomly split into training, validation, and test sets (8:1:1), with 5-fold cross-validation within the training set. Test set tumor masks were manually delineated, whereas a U-Net assisted segmentation was performed only within training to prevent data leakage. Convolutional neural network, graph convolutional network, and Transformer classifiers were compared. Class imbalance (approximately 65% vs 35%) was addressed using class weighting. Performance was evaluated using area under the receiver operating characteristic curve (AUROC) and accuracy with 95% CIs obtained by bootstrapping. AUROC differences were assessed using the DeLong test. Clinical usefulness was evaluated using decision curve analysis. Segmentation performance was quantified by Dice coefficient and intersection over union. Model interpretability was assessed using Gradient-Weighted Class Activation Mapping.

**Results:** In the MRI-only primary analysis, the Transformer achieved the best performance on the independent test set, with accuracy of 87.0% (95% CI 84.2%-89.5%) and AUROC of 0.921 (95% CI 0.901-0.945), significantly outperforming the convolutional neural network (AUROC 0.881; *P*=.02) and graph convolutional network (AUROC 0.894; *P*=.041). Sensitivity and specificity were 89.2% and 82.9%, respectively. Decision curve analysis demonstrated higher net benefit across threshold probabilities of 0.3-0.7. U-Net segmentation achieved a mean Dice coefficient of 0.892 and intersection over union of 0.814. In sensitivity analysis, CT + MRI late fusion yielded a comparable AUROC to MRI only (0.926 vs 0.921; *P*=.36), with modest incremental net benefit at higher thresholds.

**Conclusions:** In this large pre-RT imaging cohort, an MRI-driven Transformer-based DL radiomics model outperformed conventional architectures in predicting RT response in RC and demonstrated superior clinical net benefit. Late fusion of CT and MRI did not significantly improve overall discrimination but may provide incremental benefit in specific decision contexts. Multicenter external validation is warranted.

## Introduction

Rectal cancer (RC) remains one of the leading causes of cancer-related incidence and mortality worldwide, with a particularly notable rise among middle-aged and older adult populations. Radiotherapy (RT) is a cornerstone of RC management; however, its therapeutic efficacy varies substantially among patients [1]. Current clinical indicators and conventional imaging approaches show limited ability to accurately predict individual responses to RT [2], often failing to identify patients who are truly radiosensitive [3,4]. This variability poses a significant challenge in optimizing treatment strategies [2]. Consequently, there is a pressing need for robust predictive tools to guide personalized treatment and improve clinical outcomes.

The advent of radiomics has introduced the ability to extract high-dimensional quantitative features from medical images, uncovering latent biological information and offering novel insights into tumor behavior and treatment response. Unlike traditional imaging techniques, radiomics enables noninvasive prediction of therapeutic efficacy using imaging-derived biomarkers [5,6]. Nevertheless, conventional machine learning models often struggle to manage the complexity of radiomics data, particularly their high dimensionality and inherent nonlinearity [7,8]. This has led to a growing interest in the application of advanced deep learning (DL) algorithms, which offer superior capacity for feature representation and pattern recognition, to enhance the predictive power of radiomics analyses [9].

DL, particularly convolutional neural networks (CNNs), has achieved substantial advances in medical image analysis in recent years [10,11]. These models can automatically extract hierarchical features from imaging data and effectively recognize and classify complex patterns [12]. Compared with traditional approaches, DL offers notable advantages in handling high-dimensional data and performing intricate pattern recognition, making it especially well suited for analyzing radiomics data [13,14]. However, most existing studies have applied single DL algorithms in isolation, with few providing a systematic comparison of multiple models, particularly in the context of predicting RT response in patients with RC [15].

In recent years, the application of artificial intelligence (AI) in RT, cancer diagnosis, and clinical decision support has shifted from research exploration toward clinical deployment. Within the RT workflow, AI has been increasingly implemented in key steps such as organ autosegmentation, target delineation, (semi-)automated treatment planning, dose optimization, and image registration, yielding measurable time savings and improved plan consistency [16]. In the domain of cancer diagnosis, the integration of DL and radiomics has achieved substantial progress in RC lesion segmentation and feature quantification on magnetic resonance imaging (MRI). Such models can automatically localize tumor regions and characterize morphological and textural features of lesions, thereby facilitating earlier and more consistent assessment and diagnosis; this has been substantiated by both systematic reviews of RC lesion segmentation and specific studies on post–neoadjuvant chemoradiotherapy (nCRT) T2-weighted (T2W) MRI segmentation [17]. Moreover, AI models that integrate multimodal data have demonstrated practical value for personalized treatment strategies and risk prediction in clinical decision-making. For RC, a multicenter study showed that late-fusion models combining multiparametric MRI–derived DL or radiomics features with clinical variables outperformed unimodal approaches in predicting early recurrence risk and provided greater clinical net benefit as assessed by decision curve analysis (DCA) [18]. Against this background, this study integrates DL with radiomics to construct a predictive model aimed at accurately assessing the RT response in RC patients, thereby providing intelligent support for treatment optimization.

We systematically compared multiple state-of-the-art DL architectures, including CNNs, graph convolutional networks (GCNs), and Transformer models, for their ability to analyze and process radiomics imaging data in patients with RC. The Transformer architecture, in particular, exhibited superior capability in modeling complex image features through its self-attention mechanism, allowing it to effectively identify key regions relevant to RT response. By comparing these models in terms of feature extraction and predictive performance, this study investigated the potential of the Transformer model in predicting RT response in RC and evaluated its feasibility for clinical application. Unlike traditional applications of AI in tumor detection or initial diagnosis, this study focused on patients with pathologically confirmed RC. Our objective was to predict their responsiveness to RT based on pretreatment imaging, thereby enabling individualized treatment planning and minimizing unnecessary exposure to RT-related risks.

The key innovation of this study lay in the systematic application and comparison of multiple DL algorithms to enhance the accuracy of RT response prediction, with a particular emphasis on exploring the strengths of the Transformer model. By combining DL techniques with radiomics, we aimed to advance current predictive methodologies and develop a more precise model for RT response assessment. This work provides clinicians with a robust, data-driven tool to support personalized therapeutic strategies, ultimately contributing to the broader goal of precision medicine in oncology.

## Methods

### Ethical Considerations

This study was conducted in strict accordance with ethical standards. The use of imaging and clinical data was approved by the Medical Ethics Committee of Shanxi Provincial People’s Hospital. As this was a retrospective analysis, all data were obtained from routine clinical records and were fully deidentified prior to analysis. The ethics committee confirmed that additional informed consent was not required. All data were anonymized before processing, and no identifiable patient images or information was included in the manuscript or supplementary materials. Data were stored on encrypted servers with access restricted to authorized investigators. Since this study did not involve prospective data collection or patient intervention, no monetary or nonmonetary compensation was provided to participants. For any future studies involving identifiable images, written informed consent will be obtained, and the relevant materials will be submitted with the manuscript.

### Data Collection

This retrospective study included 2000 patients with RC who were treated at Shanxi Provincial People’s Hospital between January 2015 and December 2023. All patients underwent standard RT and had complete paired pretreatment computed tomography (CT) and MRI data, along with follow-up assessments of RT response.

Inclusion criteria were (1) pathologically confirmed RC, (2) receipt of standard RT, (3) availability of complete pretreatment CT and dynamic contrast–enhanced MRI (DCE-MRI) data, and (4) documented RT outcomes with follow-up records. Exclusion criteria were (1) incomplete or poor-quality imaging data, (2) missing essential clinical information or outcome labels, (3) coexisting malignancies or severe systemic diseases, and (4) patients who did not receive standard RT protocols. Treatment regimens followed the Chinese Society of Clinical Oncology guidelines for RC and primarily consisted of long-course RT (50.4 Gy in 28 fractions). Some patients also received concurrent chemotherapy and subsequent surgical intervention, depending on clinical conditions.

To ensure transparency in patient selection, a CONSORT (Consolidated Standards of Reporting Trials)-style flowchart is provided in Figure S1 in Multimedia Appendix 1, detailing the inclusion process, exclusion criteria, final sample size, and classification of patients into RT-sensitive and RT-insensitive groups.

All imaging data were acquired from routine clinical scanning systems at a single institution (Shanxi Provincial People's Hospital), encompassing MRI and CT examinations performed across different years. Detailed information regarding scanner models and acquisition parameters is provided in Table S1 in Multimedia Appendix 2.

### Data Preprocessing

Following data collection, the imaging data were preprocessed to ensure quality and consistency. The preprocessing steps included denoising, cropping, normalization, and resolution adjustment. All images were normalized to a range between 0 and 1 and resized to a resolution of 512×512 pixels. To enhance the model’s generalization ability, data augmentation techniques such as rotation, flipping, and scaling were applied to generate more diverse training samples and effectively expand the dataset. In addition, clinical data were standardized to harmonize feature values with different units and ranges, ensuring compatibility with model input requirements. The complete data collection and preprocessing workflow is illustrated in Figure S1 in Multimedia Appendix 3.

To prevent data leakage, the statistical parameters required for image normalization (mean and SD) were computed exclusively from the training set and kept fixed for both the validation and test sets. The test set was not involved in estimating normalization parameters at any stage, thereby ensuring independence and rigor in both preprocessing and model evaluation.

All DL models used in this study were trained and inferred on 2D slices; thus, the input features were derived solely from spatial texture information within single images, without relying on voxel-level 3D volume information. During preprocessing, we did not perform volumetric resampling along the *z*-axis to reconcile slice thickness differences (3 mm vs 4 mm), in order to avoid interpolation-induced artifacts that could distort 2D texture features. Because model training and prediction were conducted at the single-slice level, and all patients’ images were acquired using consistent scanning sequences and acquisition protocols, variations in slice thickness were expected to have limited impact on grayscale distribution or spatial structure of the input features. Nevertheless, we discuss the potential biases associated with differing slice thicknesses in the “Limitations” section, and we plan to explore full 3D volume resampling and the integration of 3D models in future work to further improve model robustness in cross-device settings.

### Data Annotation and Quality Control

To ensure high-quality data for model training, strict annotation and quality control procedures were applied to all imaging data and RT response labels. For the imaging side, radiologists manually delineated tumor regions, with particular attention paid to tumor boundaries and clinically relevant areas. Semiautomated annotation tools were used to assist in the delineation process. All annotations were independently reviewed by 2 associate chief radiologists (ZW and ZH), each with more than 10 years of experience. All delineation results were further validated through random sampling to ensure the consistency and accuracy of boundary recognition.

RT response labels were assigned based on posttreatment MRI evaluations and classified according to the RECIST (Response Evaluation Criteria in Solid Tumors) version 1.1. Patients who achieved a complete response (CR) or a partial response (PR) were categorized as the “good response (GR) group,” while those with stable disease or progressive disease were categorized as the “poor response group.” All imaging evaluations were performed in a blinded manner, with evaluators unaware of treatment plans and clinical outcomes. Two independent reviewers (ZW and ZH) assessed the follow-up MRIs, and interobserver agreement was assessed using the Cohen κ coefficient, which reached 0.83, indicating good consistency. Final response classifications were determined through consensus and used as ground-truth labels for supervised model training.

In particular, all tumor masks in the test set were manually annotated by experts without reference to any DL model outputs, thereby avoiding error propagation and ensuring the independence and rigor of model evaluation. The U-Net model was used exclusively on the training set to generate tumor masks as auxiliary inputs for the classification models. Its outputs were not used in any segmentation or feature extraction tasks for the test set, minimizing the risk of information leakage.

### DL Model Construction and Training

For the patient-level response prediction task, the model first performs independent inference on each 2D slice, producing a probability score indicating the likelihood of belonging to the “GR” category. Subsequently, the predicted probabilities of all slices from the same patient are averaged arithmetically to obtain an aggregated patient-level probability, and a threshold of 0.5 is applied to generate the final patient-level classification label. This aggregation strategy helps smooth slice-to-slice fluctuations caused by differences in imaging position, preventing any single atypical slice from disproportionately influencing the overall judgment. As a result, the stability and robustness of patient-level predictions are enhanced. This study used multiple DL algorithms to process radiomics imaging data and predict patient responses to RT (Figure S1 in Multimedia Appendix 4). The models compared included CNNs, GCNs, and Transformer models. All models were trained and tested on the same dataset to ensure comparability.

The Transformer model served as the primary focus due to its self-attention mechanism, which enabled the capture of complex global features and provided unique advantages in tumor region recognition. In this model, input images were divided into 16×16 patches and processed with 8 multihead attention heads and 12 Transformer encoder layers. Standard sinusoidal positional encoding was incorporated to preserve spatial structure information. The model was trained using the Adam optimizer, with an initial learning rate of 1e-4 and a cosine annealing strategy for dynamic learning rate adjustment. Training was conducted over 100 epochs, with early stopping applied if validation loss did not decrease for 10 consecutive epochs.

As the comparative baseline, the CNN used a residual networks (ResNet-34) architecture, consisting of 34 residual convolutional layers organized into 4 residual stages with channel dimensions of 64, 128, 256, and 512, respectively. Feature extraction was performed on 2D patches of the input images. The output of the final global average pooling layer was fed into a 2-layer fully connected classification head (hidden layer size=256, activation function=ReLU) to generate predictions of RT response.

The GCN module adopted a 3-layer GCNConv structure, where nodes represented embedded features of image patches or regions of interest (ROIs). The adjacency matrix was constructed based on spatial proximity using a k-nearest neighbors approach (k=8). Each layer used a ReLU activation function, and dropout (*P*=.30) was applied between layers to mitigate overfitting. The aggregated node features after global pooling were passed to a classification layer. All models were trained under identical conditions, using the same loss function (weighted cross-entropy), optimizer (Adam, learning rate=1×10^–^⁴), and training strategy (cosine annealing schedule with early stopping) to ensure a fair performance comparison. Hyperparameter tuning and L2 regularization were applied throughout training to reduce overfitting risks. The final model was independently evaluated on the test set and compared against other networks, with particular emphasis on its performance in feature extraction and classification accuracy.

Given the class imbalance in RT response labels (approximately 65% of patients in the GR group and 35% in the poor response group), a class weighting strategy was introduced during training. Specifically, inverse-frequency weights based on sample distribution were applied in the loss function to enhance the model’s sensitivity to the minority class (poor response patients). This approach effectively mitigated the prediction bias caused by unequal class distribution and yielded higher sensitivity and overall stability in the validation set.

In the final implementation of this study, the Transformer architecture was used solely as an image-level classifier. The network consisted of multiple encoder layers and a classification head, without any decoder branch or explicit segmentation output (eg, a “Seg Head”). The tumor segmentation task was performed entirely by an independent U-Net model, and the 2 models operated independently throughout the training and evaluation processes.

### Dataset Splitting and Cross-Validation

To evaluate the generalization ability of the models, the dataset was divided into training, validation, and test sets in a ratio of 8:1:1. Random sampling was used to ensure the representativeness of each subset. The 8:1:1 split was chosen based on the sufficiently large sample size (2000 patients), which supported the high-dimensional feature extraction needs of DL models. A larger training set enabled the model to better learn complex nonlinear patterns, while 200 patients each were retained for independent validation and testing. To further improve model stability and generalizability, 5-fold cross-validation was applied during training, with k set to 5. Stratified sampling was used to preserve consistent class distributions across all folds. All models were trained and evaluated on the same data splits, and the average performance metrics across the 5 folds were reported as the final results. This approach ensured robust evaluation, minimized bias, and enhanced the reproducibility of model performance across different subsets.

To evaluate model generalizability, the dataset was divided into training, validation, and testing subsets in an 8:1:1 ratio. All partitions were performed at the patient level, ensuring that all images, slices, and augmented samples from the same patient were assigned exclusively to a single subset to prevent information leakage. Stratified random sampling was applied to maintain consistent class distribution across subsets. To further enhance model robustness and generalization performance, 5-fold cross-validation (k=5) was conducted within the training cohort, also based on patient-level partitioning. This approach ensured the independence of the validation data and the reproducibility of the experimental results.

To avoid data leakage, data augmentation in this study was applied exclusively to the training set, while no augmentation was performed on the validation or test sets. All augmented samples were strictly linked to their original patients and existed only within the training subset, ensuring that none entered the independent test set. Consequently, each test-set patient corresponded to exactly 1 nonaugmented imaging input during inference, which explains why the total count in the confusion matrix matches the original number of patients (n=200).

### Model Evaluation and Performance Analysis

Model performance was evaluated using multiple classification metrics, including accuracy, sensitivity, specificity, receiver operating characteristic (ROC) curves, area under the curve (AUC) values, and confusion matrices, to comprehensively assess the predictive accuracy of RT response in RC. Ninety-five percent CIs were calculated for all metrics to strengthen the robustness and interpretability of results.

To statistically compare model performances and verify the relative advantage of the Transformer model, pairwise bootstrap resampling (10,000 iterations) and DeLong’s test were performed for AUC comparisons, with *P* values reported. ROC curves and their confidence intervals were used to visually and quantitatively compare the classification performance of different models.

Calibration was evaluated on the independent test set by plotting a calibration curve (reliability diagram) using 10 equal-frequency bins (deciles). For each bin, we reported the mean predicted probability and the observed event rate. Overall calibration was quantified with the Brier score (the mean squared error between predicted probabilities and true labels). All calibration analyses were performed once on the test set without any retraining or post hoc recalibration to avoid information leakage. To ensure fairness, the bin boundaries and the evaluation pipeline were kept identical across input settings (MRI-only vs CT + MRI).

During evaluation, particular emphasis was given to evaluating the Transformer model’s performance under varying segmentation input conditions. Its prediction results were further analyzed through Gradient-Weighted Class Activation Mapping (Grad-CAM) visualization to interpret attention regions and their clinical relevance. Tumor segmentation performance using the U-Net model was evaluated on the independent test set, with the Dice Similarity Coefficient (DSC) and the Intersection over Union (IoU) used as primary metrics. Manually delineated tumor regions, confirmed by 2 experienced radiologists, served as the gold standard. Failure cases were manually analyzed to identify potential factors contributing to segmentation inaccuracies, such as blurred tumor boundaries, low tissue contrast, or irregular tumor morphology. Detailed segmentation outcomes are shown in the “Results” section, and representative case images are provided in multimedia appendices.

### Image Data Analysis Method

The image data analysis pipeline in this study consisted of 2 independent stages: tumor segmentation and image-level classification. First, a U-Net segmentation network was used to delineate tumor regions on the raw MRI or CT images, generating binary masks (Figure S1 in Multimedia Appendix 5). This segmentation model served solely to provide ROIs and did not participate in subsequent classification learning. Next, image patches or ROI features extracted from the masked tumor regions were fed into 3 types of classification models, CNNs, GCNs, and a Transformer classifier (Figure S1 in Multimedia Appendix 6), to predict patients’ RT response.

It is important to emphasize that the Transformer model used in this study was a pure classification architecture composed only of an encoder and a multilayer perceptron classification head. It did not include any decoder structure, upsampling pathway, or segmentation outputs. Accordingly, it was entirely independent of the U-Net segmentation model used to generate masks; the 2 models shared no parameters and had no information exchange during training or inference. The GCN was used to model spatial relationships within the tumor region, further enhancing classification performance. The overall model evaluation and performance analysis workflow is shown in Figure S1 in Multimedia Appendix 7.

### Input Modalities and Multimodal Fusion Strategy

In the primary analysis, the classifier used MRI-only inputs—pre-RT DCE-MRI and T2W sequences—and extracted features or patches or embeddings strictly within expert-annotated tumor masks, thereby minimizing bias from cross-modal acquisition heterogeneity and registration error. CT was used solely for quality control and, when necessary, rigid registration and was not provided to the classifier. Per reviewer request, we additionally implemented late fusion of CT + MRI under the same data split, loss function, class weighting, and hyperparameter settings as the primary analysis. Specifically, patient-level representations were extracted from CT and MRI with identical backbone encoders; after feature alignment and normalization, the representations were concatenated immediately before the classification head and trained or evaluated jointly (all other procedures unchanged). To ensure a fair comparison, the independent test set was kept fixed (n=200; good responders, n=130 and poor responders, n=70); only the input modality configuration was varied.

### Statistical Analysis

This study applied various DL techniques to analyze radiomics imaging data from patients with RC. Initially, CNNs were used to extract features from the image data, capturing fine-grained details of tumor tissues. During image segmentation, deep segmentation networks such as U-Net and Mask R-CNN were used to identify and annotate tumor regions, ensuring accurate localization of key lesions. The self-attention mechanism of the Transformer model was used to manage complex spatial relationships, thereby improving the model’s pattern recognition capabilities. GCNs were also applied to extract global and local spatial features from the images, effectively modeling both tumor and peritumoral tissue characteristics. By integrating multiple DL models, the study aimed to achieve accurate predictions of RT response and support personalized treatment recommendations.

During model evaluation, various statistical methods were used to comprehensively assess predictive performance. Metrics, including sensitivity, specificity, precision, and recall, were calculated to evaluate classification outcomes across different categories. Confusion matrices were plotted to visualize model performance in distinguishing between positive and negative RT responses. To assess predictive ability across varying thresholds, ROC curves and AUC values were generated for each model. These statistical analyses facilitated a detailed comparison of the strengths and limitations of each DL approach in predicting RT response in patients with RC.

## Results

### Dataset Sample Distribution Balance and Generalization Ability

In this study, radiomic imaging data from 2000 patients with RC were collected and randomly divided into training (1600 cases), validation (200 cases), and test (200 cases) sets using an 8:1:1 ratio. Approximately 65% (1300/2000) of patients exhibited a favorable response to RT and were classified as the “sensitive” group, while the remaining 35% (700/2000) were categorized as “nonsensitive.” As shown in Table 1, we further compared baseline demographic and clinical characteristics—including age, sex, T stage, N stage, tumor location, and carcinoembryonic antigen levels—between the training and test sets. None of the variables showed statistically significant differences (*P*>.05). These results indicate that the random partitioning of the dataset was unbiased and that the training and test sets were highly comparable. This balanced distribution provides a reliable foundation for model training and independent validation, thereby enhancing the generalizability and robustness of the proposed approach.

**Table 1.** Baseline demographic and clinical characteristics of patients in the training and testing cohorts^a^.

Variable	Training cohort (N=1600)	Testing cohort (N=200)	*P* value
Age (years), mean (SD)	58.7 (10.9)	59.1 (11.2)	.62
Sex, n (%)			.53
Male	980 (61.3)	118 (59.0)	
Female	620 (38.7)	82 (41.0)	
T stage, n (%)			.78
T2	430 (26.9)	50 (25.0)	
T3	930 (58.1)	120 (60.0)	
T4	240 (15.0)	30 (15.0)	
N stage, n (%)			.67
N0	610 (38.1)	80 (40.0)	
N1	710 (44.4)	86 (43.0)	
N2	280 (17.5)	34 (17.0)	
Tumor location, n (%)			.79
Upper rectum	310 (19.4)	36 (18.0)	
Midrectum	860 (53.8)	112 (56.0)	
Lower rectum	430 (26.9)	52 (26.0)	
CEA^b^ level (ng/mL), median (IQR)	3.6 (2.1-6.8)	3.8 (2.2-7.0)	.73
Response category, n (%)			>.99
Good response	1040 (65.0)	130 (65.0)	
Poor response	560 (35.0)	70 (35.0)	

a This table shows the baseline differences between the training and test sets in demographic characteristics (age and sex), tumor clinical staging (T stage and N stage), tumor location, and CEA level. Statistical comparisons were performed using independent-samples *t *tests, chi-square tests, or Mann-Whitney *U* tests, as appropriate. All *P* values were greater than .05, indicating no significant differences between the training and test sets. This demonstrates that the random allocation process was unbiased and that the cohorts were well matched and comparable.

b CEA: carcinoembryonic antigen.

### High Accuracy of U-Net Model in Tumor Region Segmentation

**Figure 1.**
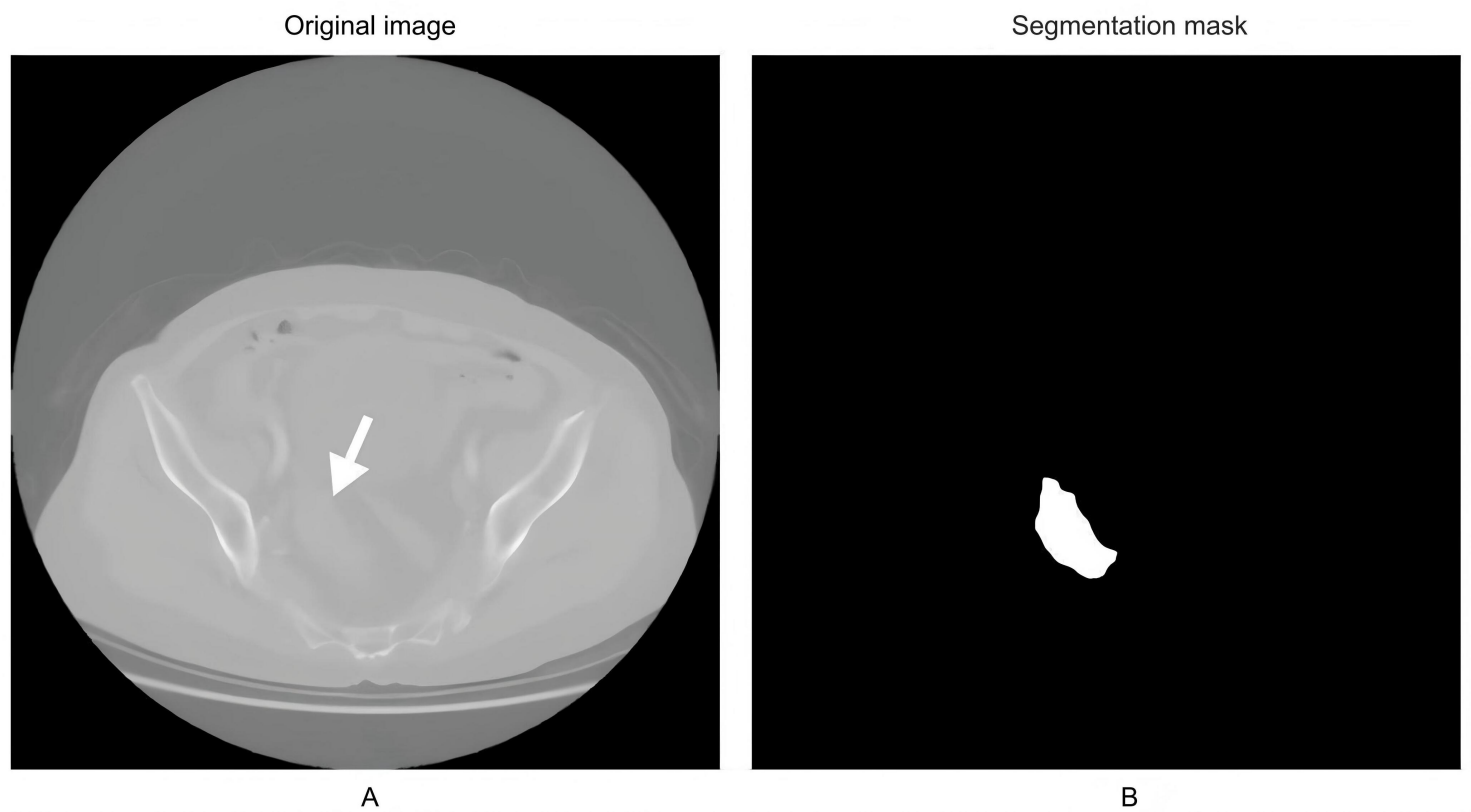
Tumor region segmentation results. (**A**) Original image. The white arrow indicates the rectal tumor lesion. (**B**) Model segmentation result image (tumor region highlighted).

In this study, a U-Net segmentation network was applied to automatically segment tumor regions in the imaging data of patients with RC. The results demonstrated excellent performance of the model in tumor recognition and segmentation tasks. On the independent test set, the U-Net model achieved a mean DSC of 0.892 (95% CI 0.881‐0.904) and a mean IoU of 0.814 (95% CI 0.800‐0.829), indicating high segmentation consistency and robustness. The model precisely delineated tumor boundaries and clearly separated tumor regions from surrounding structures. The original image is shown in Figure 1A, and the corresponding segmentation mask generated by the U-Net model is shown in Figure 1B.

When compared with manually annotated gold-standard segmentations by expert radiologists, the model showed a high degree of agreement in both boundary definition and tumor morphology. Notably, the U-Net model maintained strong performance even when segmenting tumors with irregular shapes or blurred margins. However, a small number of inaccurate segmentations were observed, primarily in cases characterized by indistinct tumor margins, low contrast with adjacent tissues, or atypical tumor morphology. These challenging cases provide directions for future model optimization.

### Outstanding Performance of the Transformer Model in Predicting RT Response

**Figure 2.**
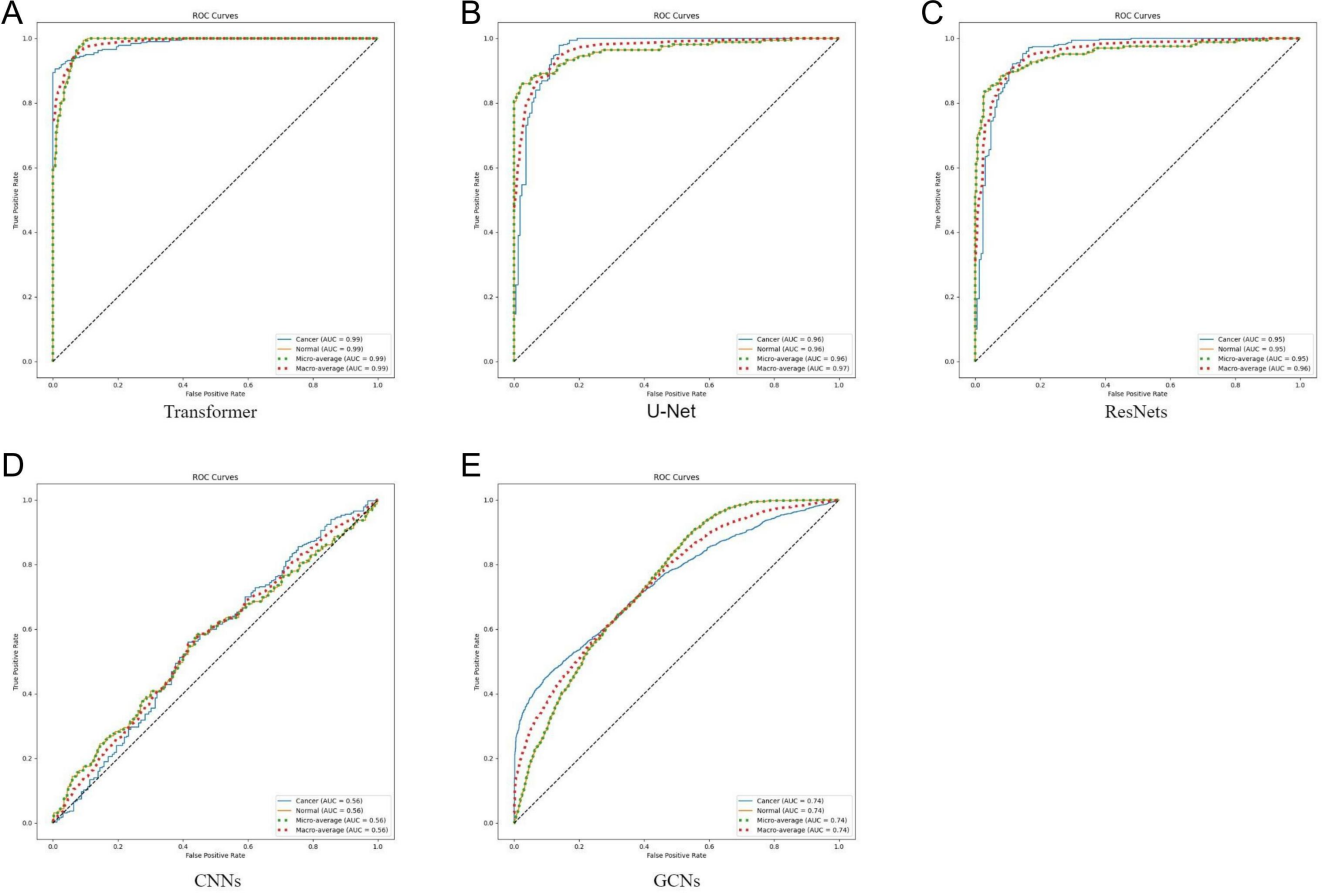
ROC curve comparison of different models. (A) Transformer, (B) U-Net, (C) ResNet, (D) CNN, and (E) GCN. AUC: area under the curve; CNN: convolutional neural network; GCN: graph convolutional network; ROC: receiver operating characteristic.

This study compared the performance of several DL models in predicting the RT response in patients with RC. The results showed that the Transformer model achieved the highest overall performance, with an accuracy of 87%. As illustrated in Figure 2A, the Transformer leveraged its self-attention mechanism to effectively process complex patterns and fine-grained features within the radiomics imaging data, offering a distinct advantage in capturing global contextual information. In comparison, the CNN (Figure 2D) and GCN (Figure 2E) models achieved accuracies of 82% and 84%, respectively, both lower than that of the Transformer model. Analysis of ROC curves (Figures 2A-2E) further highlighted the Transformer model’s superior discriminative ability, as reflected by its AUC value of 0.921 (95% CI 0.901‐0.945), which exceeded that of the CNN (Figure 2D) and GCN (Figure 2E).

To enhance the robustness of the results, 95% CIs were calculated for all classification metrics across the models (Table 2). On the independent test set, the Transformer model achieved an accuracy of 87.0% (95% CI 84.2%‐89.5%), sensitivity of 89.2% (95% CI 85.3%‐92.4%), specificity of 82.9% (95% CI 79.2%‐88.1%), and an AUC of 0.921 (95% CI 0.901‐0.945). In contrast, the CNN model reached an AUC of 0.881 (95% CI 0.854‐0.906), while the GCN model achieved an AUC of 0.894 (95% CI 0.869‐0.918).

**Table 2.** Performance comparison of deep learning models in predicting radiotherapy response^a^.

Model	Accuracy	Sensitivity	Specificity	AUC^b^	*P* value vs Transformer
Transformer	87.0% (84.2%-89.5%)	89.2% (85.3%-92.4%)	82.9% (79.2%-88.1%)	0.921% (0.901%-0.945%)	—^c^
CNN^d^	82.0% (78.9%-85.0%)	83.7% (79.4%-87.6%)	80.2% (75.0%-84.6%)	0.881% (0.854%-0.906%)	.02
GCN^e^	84.0% (80.4%-87.2%)	85.9% (81.7%-89.7%)	82.1% (77.0%-86.4%)	0.894% (0.869%-0.918%)	.041

a This table compares the performance of 3 deep learning models (transformer, CNN, and GCN) in predicting radiotherapy response in rectal cancer using an independent test set. All metrics are reported as mean values with 95% CI. The primary evaluation metrics include accuracy, sensitivity, specificity, and the area under the curve (AUC). Differences in AUC were assessed using the DeLong test. The results demonstrate that the Transformer model outperformed both CNN and GCN models across all evaluation metrics, with the differences in AUC reaching statistical significance (*P*<.05).

b AUC: area under the curve.

c Not available.

d CNN: convolutional neural network.

e GCN: graph convolutional network.

To evaluate the statistical significance of AUC differences between the Transformer and other models, pairwise bootstrap resampling (10,000 iterations) and DeLong’s test were conducted. The results showed that the Transformer model significantly outperformed both the CNN model (*P*=.02) and the GCN model (*P*=.041) in terms of AUC. These findings indicated that the Transformer model provided a statistically significant advantage in identifying imaging features associated with RT response.

### Confusion Matrix Reveals the Classification Capability of the Transformer Model

**Figure 3.**
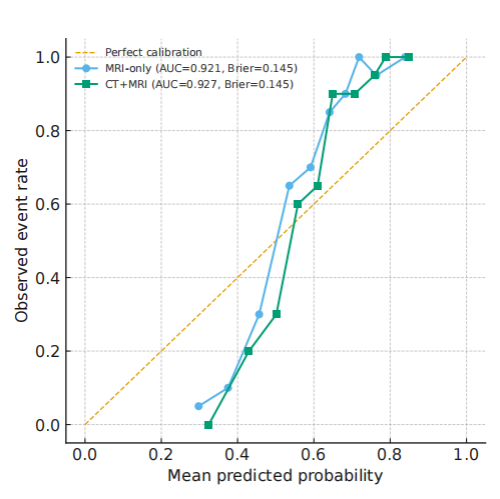
Calibration of the Transformer classifier on the independent test set. Reliability curves of the Transformer classifier on the independent test set (n=200) using 10 equal-frequency bins. The dashed line indicates perfect calibration. Curves are shown for both MRI-only and CT + MRI input settings, with the corresponding AUC and Brier score reported within parentheses. AUC: area under the curve; CT: computed tomography; MRI: magnetic resonance imaging.

To further assess classification performance, a confusion matrix was constructed to evaluate the Transformer model’s predictive ability. As shown in Table 3, among the 200 patients in the test set, the model correctly identified 89% of RT-sensitive patients. The false-positive rate, representing RT-insensitive patients incorrectly classified as sensitive, was only 8%. These results further validated the model’s effectiveness across both classes, particularly in correctly identifying RT-insensitive patients. The Transformer model significantly reduced misclassification errors, a critical factor in clinical decision-making. Accurate identification of RT-insensitive patients was essential for enabling timely adjustments to treatment plans, avoiding ineffective RT, and ultimately improving patient prognosis and survival outcomes. On the independent test set, the Transformer’s calibration curve closely followed the ideal 45° line, with only mild overconfidence observed in the high–predicted-probability region. The corresponding Brier scores were identical for both input settings: MRI-only, 0.145; and CT + MRI (late fusion), 0.145. Overall calibration was therefore comparable between modalities. Taken together with the AUCs (0.921 vs 0.927) and the DCA results, adding CT did not yield a meaningful improvement in global performance, but it produced a slight and consistent gain in net benefit within higher decision-threshold ranges (Figure 3 and Table 4).

**Table 3.** Confusion matrix results for the Transformer model^a^.

Category	Sensitive class (good response to radiotherapy)	Insensitive class (poor response to radiotherapy)
Predicted as sensitive class	116 (true positive)	12 (false positive)
Predicted as insensitive class	14 (false negative)	58 (true negative)

a This table shows the confusion matrix of the Transformer model on the independent test set (magnetic resonance imaging–only, n=200). True positives represent the number of patients correctly predicted as radiotherapy-sensitive (116 cases); false positives represent the number of patients incorrectly predicted as sensitive but actually nonsensitive (12 cases); false negatives represent the number of patients incorrectly predicted as nonsensitive but actually sensitive (14 cases); and true negatives represent the number of patients correctly predicted as radiotherapy nonsensitive (58 cases). Based on this matrix, the calculated performance metrics were as follows: accuracy=87.0%, sensitivity=89.2%, and specificity=82.9%.

**Table 4.** Calibration results of the Transformer model under MRI^a^-only and CT^b^ + MRI settings^c^.

Setting	Bin	Count	pred_mean	obs_rate	pred_min	pred_max
MRI-only	1	20	0.297790855	0.05	0.182508038	0.338796612
MRI-only	2	20	0.374815574	0.1	0.350143687	0.407613655
MRI-only	3	20	0.456552744	0.3	0.411604379	0.488843116
MRI-only	4	20	0.535671487	0.65	0.489127784	0.56889754
MRI-only	5	20	0.591075985	0.7	0.573348236	0.619675996
MRI-only	6	20	0.641167999	0.85	0.620093517	0.662707158
MRI-only	7	20	0.682686695	0.9	0.663585095	0.697369603
MRI-only	8	20	0.718369677	1	0.697816083	0.742269577
MRI-only	9	20	0.763478434	0.95	0.742374832	0.788465779
MRI-only	10	20	0.839248874	1	0.789370839	0.919921412
CT + MRI	1	20	0.324232379	0	0.240873315	0.387211357
CT + MRI	2	20	0.429795288	0.2	0.389460186	0.473106637
CT + MRI	3	20	0.501537256	0.3	0.473698194	0.538277464
CT + MRI	4	20	0.558194525	0.6	0.538663984	0.585177755
CT + MRI	5	20	0.609770763	0.65	0.586338258	0.625575137
CT + MRI	6	20	0.649248771	0.9	0.626019055	0.677019798
CT + MRI	7	20	0.707037992	0.9	0.683165825	0.735004007
CT + MRI	8	20	0.758477804	0.95	0.737737455	0.775617046
CT + MRI	9	20	0.788180535	1	0.7760233	0.80785209
CT + MRI	10	20	0.847920043	1	0.808998069	0.928076363

a MRI: magnetic resonance imaging.

b CT: computed tomography.

c This table shows the calibration binning results of the transformer model on the independent test set under 2 input settings: MRI-only and CT + MRI. The predicted probabilities (pred_mean) were divided into 10 equal-frequency bins (bin), each containing 20 samples. Obs_rate represents the observed positive rate (proportion of radiotherapy-sensitive patients), while pred_min and pred_max indicate the minimum and maximum predicted probabilities within each bin, respectively. The results show that both input settings exhibit calibration trends close to the ideal diagonal, indicating good agreement between predicted probabilities and observed rates. Notably, the CT + MRI setting demonstrated a slight improvement in net benefit within the higher threshold range.

### Grad-CAM Visualization to Validate the Accuracy of Feature Extraction

**Figure 4.**
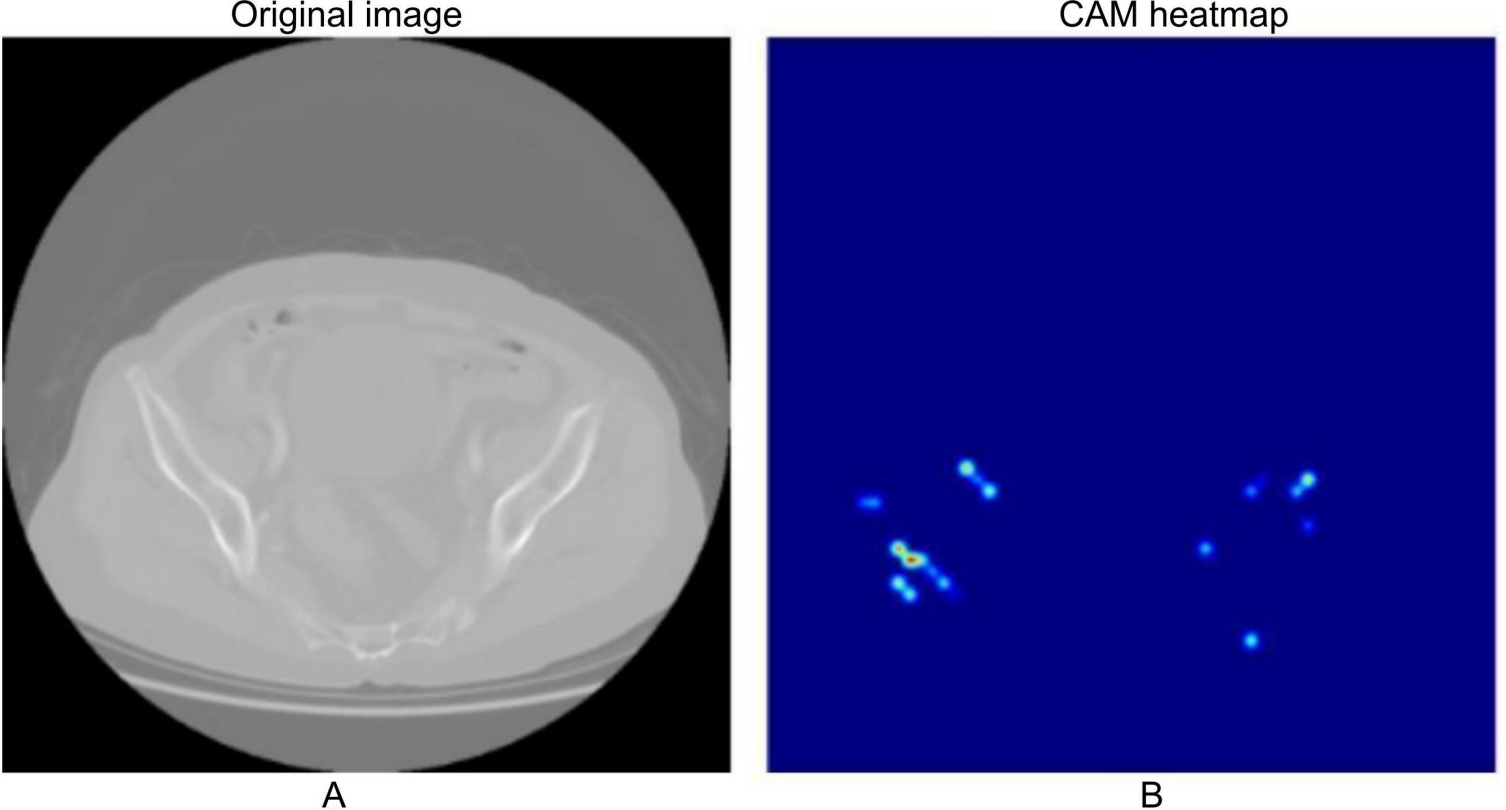
Gradient-Weighted Class Activation Mapping (Grad-CAM) visualization of feature extraction in the Transformer model. (A) Original image. (B) Grad-CAM visualization image: displays the areas where the model focuses, particularly on tumor edges and high-density regions. Red areas represent the regions of high attention by the model. CAM: Class Activation Mapping.

The effectiveness of the Transformer model was reflected not only in its overall predictive performance but also in the depth and precision of its feature extraction. Grad-CAM was used to visualize the model’s feature extraction process. The results revealed that the model primarily focused on tumor boundaries and high-density regions within the tumor. As shown in Figures 4A and 4B, the model was particularly adept at capturing complex structures in heterogeneous tumor areas and using them to predict RT response. The visualization results further demonstrated that the model effectively filtered out irrelevant background information and concentrated on diagnostically significant regions. This selective focus enhanced both the classification accuracy and the clinical interpretability of the model. The ability to highlight meaningful anatomical and pathological features supported the Transformer model as a reliable diagnostic aid for clinicians in treatment planning and response prediction.

### Hyperparameter Optimization and Model Iteration Analysis

**Figure 5.**
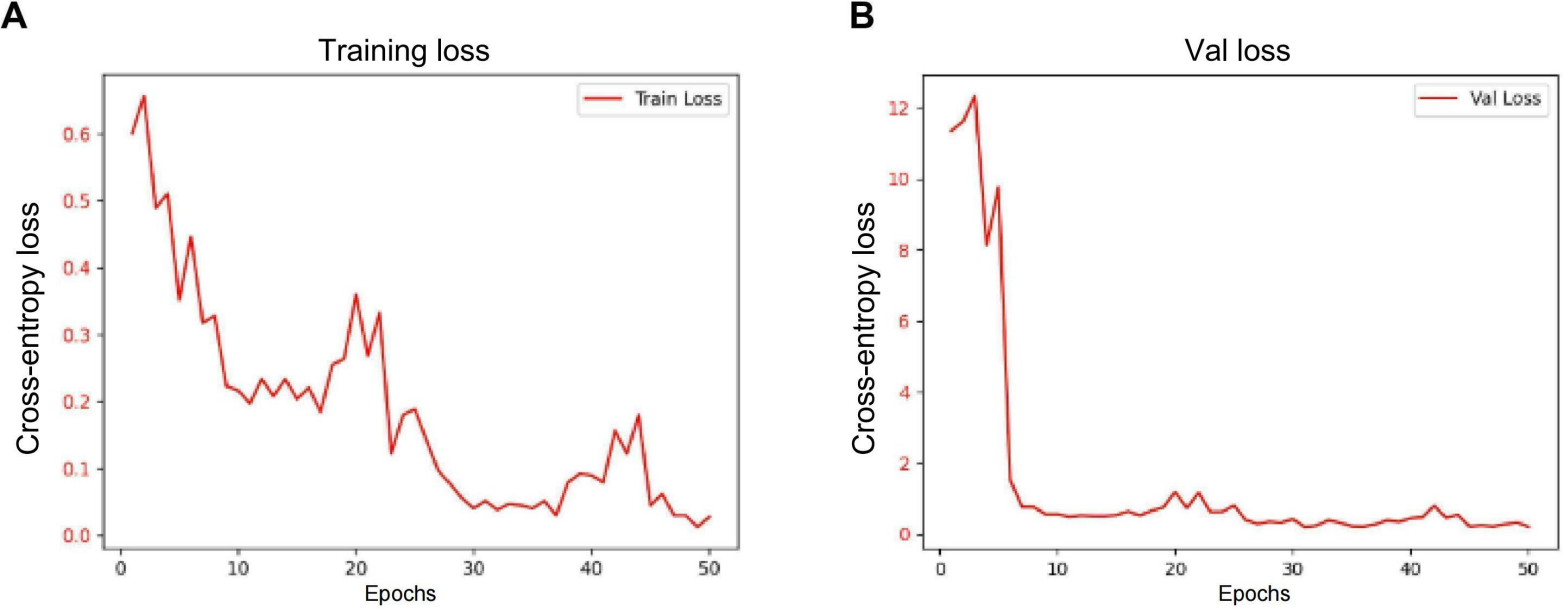
Changes in loss and accuracy for training and validation sets. (A) Loss curve: the loss values for both the training and validation sets decrease gradually over 50 epochs. (B) The validation loss curve (Val loss) indicates that the model stabilized during the later training phase without signs of overfitting.

During model training and optimization, adjustments to hyperparameters such as learning rate, batch size, and regularization substantially improved the performance of the Transformer model. After 50 training epochs, the validation loss steadily decreased to 0.01, indicating good model convergence and stable training dynamics. As training progressed, the loss curves of the training and validation sets gradually converged, with no apparent signs of overfitting, further confirming the critical role of hyperparameter optimization in enhancing model performance (see Figure 5A for training loss and Figure 5B for validation loss). The model maintained consistently low validation loss and favorable convergence characteristics, establishing a solid foundation for subsequent testing and external validation.

### Clinical Benefit Analysis Based on DCA

**Figure 6.**
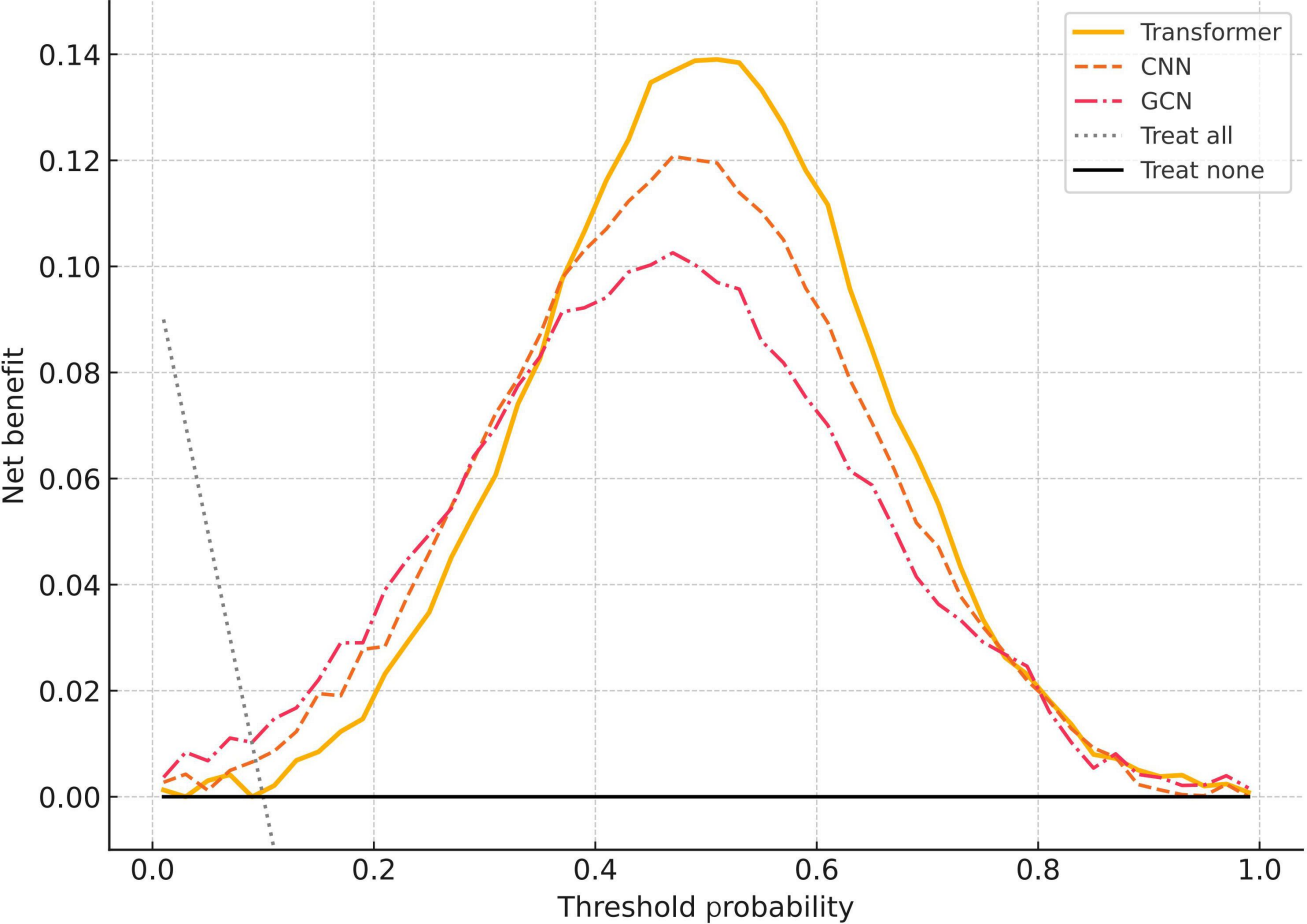
Decision curve analysis. The figure shows the comparison of net benefit across different probability thresholds for the Transformer, CNN, and GCN models. The dashed lines represent the “treat all” and “treat none” strategies, respectively. The Transformer model demonstrated higher net benefits within the primary clinical decision-making range of 0.3‐0.7, highlighting its clinical use in predicting radiotherapy sensitivity. CNN: convolutional neural network; GCN: graph convolutional network.

To further evaluate the potential clinical use of the DL models in RT decision-making, DCA was performed to compare the net benefit of the Transformer, CNN, and GCN models across different probability thresholds (Figure 6). The results showed that across most threshold ranges—particularly between 0.3 and 0.7—the Transformer model achieved a higher net benefit than both the “treat all” and “treat none” strategies, as well as outperforming the CNN and GCN models. This suggests that the Transformer model provides greater decision support capacity in clinical practice.

For example, when predicting RT-sensitive patients, the Transformer model maintained a high true-positive rate while significantly reducing the likelihood of unnecessary RT and associated side effects. These findings highlight the model’s potential as a valuable tool for individualized RT decision-making.

### Sensitivity Analysis of Imaging Input Settings (MRI-Only Versus CT + MRI)

To quantify the incremental value of CT, we compared MRI-only with late-fusion CT + MRI under identical training or validation procedures (same splits, loss, class weighting, and hyperparameters). Using the Transformer as an exemplar, overall discrimination on the independent test set was similar between modalities: Transformer (MRI-only): accuracy 87.0%, AUC 0.921 (95% CI 0.901‐0.945), sensitivity 89.2% (true positive 116/130), specificity 82.9% (true negative 58/70), Brier score 0.145, and area under the precision–recall curve 0.900; Transformer (CT + MRI): accuracy 87.5%, AUC 0.926 (95% CI 0.905‐0.948), sensitivity 90.0% (true positive 117/130), specificity 82.9% (true negative 58/70), Brier score 0.142, and area under the precision–recall curve 0.905.

DeLong’s test indicated no significant difference in AUC between the 2 settings (*P*=.36). In DCA, CT + MRI yielded a slight and consistent gain in net benefit at higher threshold probabilities (>0.6), whereas curves largely overlapped within the primary clinical range (0.3‐0.7). Overall, given a unified MRI acquisition protocol in this single-center cohort, MRI-only achieved robust performance; adding CT did not confer a statistically significant global improvement but may offer marginal clinical value at specific, higher decision thresholds. For completeness, test-set AUCs (95% CI) across all 3 architectures (CNN, GCN, and Transformer) under both input settings are summarized in Table 5, and full classification metrics for the Transformer are provided in Table 6.

**Table 5.** Comparison of the Transformer model performance under different input settings^a^.

Input setting	Accuracy	AUC	95% CI (AUC^b^)	Sensitivity	Specificity	Brier	AUCPR^c^	DeLong test *P* value
MRI^d^-only	87.00%	0.921	0.901‐0.945	89.2% (116/130)	82.9% (58/70)	0.145	0.9	—^e^
CT + MRI (late fusion)	87.50%	0.926	0.905‐0.948	90.0% (117/130)	82.9% (58/70)	0.142	0.905	.36

a This table compares the predictive performance of the Transformer model under 2 input settings: magnetic resonance imaging (MRI)–only and CT + MRI (late fusion). The main evaluation metrics include accuracy, area under the curve (AUC) with its 95% CI, sensitivity, specificity, Brier score, area under the precision–recall curve (AUCPR), and the DeLong test *P *value for AUC comparison. The results indicate that the overall discriminative performance of the 2 input settings was comparable (AUC difference, *P*=.36). The CT + MRI configuration showed slightly better Brier score and AUCPR at higher threshold ranges, suggesting a minor net benefit under certain clinical decision thresholds.

b AUC: area under the curve.

c AUCPR: area under the precision–recall curve.

d MRI: magnetic resonance imaging.

e Not available.

**Table 6.** Comparison of AUC^a^ values across architectures under MRI^b^-only and CT^c^ + MRI settings^d^.

Architecture	MRI-only AUC (95% CI)	CT + MRI AUC (95% CI)	DeLong test *P* value
CNN^e^	0.881 (0.854‐0.906)	0.888 (0.861‐0.912)	.22
GCN^f^	0.894 (0.869‐0.918)	0.899 (0.873‐0.922)	.29
Transformer	0.921 (0.901‐0.945)	0.926 (0.905‐0.948)	.36

a AUC: area under the curve.

b MRI: magnetic resonance imaging.

c CT: computed tomography.

d This table compares the area under the curve (AUC) performance and corresponding 95% CI of 3 deep learning models—CNN, GCN, and Transformer—under 2 input conditions: MRI-only and CT + MRI. Differences in AUC were evaluated using the DeLong test. The results show that all 3 models exhibited slight improvements in AUC under the CT + MRI condition; however, none of the differences reached statistical significance (*P*>.05). Among the 3, the Transformer model achieved the highest AUC in both settings (MRI-only: 0.921; CT + MRI: 0.926).

e CNN: convolutional neural network.

f GCN: graph convolutional network.

### Computational Efficiency and Inference Time Analysis

To quantitatively evaluate the computational overhead and deployment feasibility of different models in clinical scenarios, we further compared CNNs, GCNs, and Transformer-based models in terms of parameter count, theoretical computational complexity (Floating-Point Operations [FLOPs]), and inference time on an independent test set. Inference-time evaluation was conducted under a fixed hardware configuration (GPU: NVIDIA RTX 3090, 24 GB memory), using FP32 precision, with synchronized timing performed after model warm-up. The timing scope included model forward inference and patient-level probability aggregation (ie, arithmetic averaging of predicted probabilities across all 2D slices belonging to the same patient), while offline image preprocessing and data-loading overheads were excluded.

As summarized in Table 7, the Transformer model exhibits a larger parameter size (86.4 M) and higher FLOPs (17.8 G) than the CNN (ResNet-34) and GCN architectures, reflecting its increased model complexity. Nevertheless, on the test set, the Transformer model achieves an average per-slice inference time of 3.8 milliseconds. At the patient level (median number of slices: 20, IQR 16‐24), the overall inference time is 78 (IQR 63-95) milliseconds per patient, which remains well within the acceptable range for real-time clinical decision support. By comparison, the patient-level inference times of the CNN and GCN models are 42 milliseconds and 55 milliseconds, respectively.

**Table 7.** Computational efficiency and inference latency of the evaluated models^a^.

Model	Parameters, millions	FLOPs^b^, billions	Inference time per slice (ms), mean (SD)	Average slices per patient, median (IQR)	Inference time per patient (ms)
CNN^c^ (ResNet-34)	21.8	3.6	2.1 (0.3)	20 (16-24)	42 (34‐50)
GCN^d^	28.5	5.2	2.7 (0.4)	20 (16-24)	55 (44‐66)
Transformer	86.4	17.8	3.8 (0.5)	20 (16-24)	78 (63‐95)

a Parameter counts and FLOPs were calculated based on a single region of interest input. Inference time was measured on an independent test set (n=200) and includes model forward inference as well as patient-level prediction probability aggregation, while offline image preprocessing and data-loading overheads were excluded. All results were obtained under identical hardware and software environments.

b FLOPs: Floating-Point Operations.

c CNN: convolutional neural network.

d GCN: graph convolutional network.

These results indicate that although the Transformer architecture incurs a higher computational cost than conventional convolutional models, its inference latency remains low. Moreover, when weighed against its substantial advantages in predictive performance and clinical net benefit, the increased computational overhead is acceptable for practical clinical applications.

## Discussion

### Principal Findings

In this study, a DL-based radiomics model was developed to predict RT response in patients with RC. A systematic comparison of multiple models, including CNNs, GCNs, and Transformers, revealed that the Transformer architecture demonstrated the best overall performance. By leveraging its self-attention mechanism, the Transformer effectively captured complex imaging features within key tumor regions, achieving an accuracy of 87% and an AUC of 0.921 on the test set. In addition to its predictive accuracy, the Transformer model demonstrated strong interpretability, as confirmed by Grad-CAM visualization, which highlighted its focus on clinically relevant tumor regions. These findings underscore the model’s potential as a reliable and interpretable tool for supporting individualized RT planning in clinical settings.

During training, 5-fold cross-validation (k=5) was adopted based on the following considerations. First, it balanced statistical stability and sample efficiency. In this cohort (n=2000; class ratio ≈65%/35%), k=5 ensured approximately 200 validation cases per fold, providing sufficient positive and negative samples to stably estimate AUC, calibration, and DCA metrics. Larger k would yield smaller validation subsets, increasing variability in calibration and DCA estimates, whereas smaller k would raise estimation bias and reduce the resolution of hyperparameter selection. Second, it accounted for computational cost and practical reproducibility: compared with 10-fold cross-validation, 5 folds substantially reduced training time and resource requirements, making replication in a clinical research setting more feasible. To assess robustness, we conducted sensitivity analyses using k=3 and k=10 and repeated 5-fold cross-validation with different random seeds under the same patient-level stratification. Across all settings, the relative ranking of the Transformer and baseline models remained consistent, fold-to-fold AUC variability was comparable with the main analysis, and no systematic instability or overfitting was observed. Compared with 5 folds, 10 folds primarily increased training time and triggered more frequent early stopping without materially affecting final test performance. Three-folds showed slightly greater variance in validation metrics and reduced hyperparameter discriminability. In summary, k=5 provided an optimal balance among performance stability, computational feasibility, and methodological rigor. To avoid information leakage, cross-validation was performed exclusively within the training set using stratified, patient-level splits; all images, slices, and augmented samples from the same patient were confined to a single fold. The independent test set was reserved for a one-time final evaluation only.

### Comparison With Prior Work

RT remains a cornerstone in RC treatment; however, predicting individual response to RT continues to be a major clinical challenge due to significant patient heterogeneity [19]. Radiomics, an emerging technology capable of extracting high-dimensional features from medical images, has opened new avenues for predicting treatment response [20]. However, traditional machine learning approaches have shown limited effectiveness in modeling the high-dimensional, heterogeneous nature of radiomics data [21]. The advent of DL, particularly CNNs, has markedly improved pattern recognition capabilities in this domain [7,9,22]. Unlike prior studies that applied single-model approaches, our work systematically compared 3 advanced DL architectures and identified the Transformer as the most effective model for complex image-based RT response prediction [23].

Several recent studies have attempted to integrate DL into tumor-imaging analysis and RT response prediction. For instance, Xu et al [24] applied CNNs for breast region segmentation using DCE-MRI in breast cancer, demonstrating strong performance in local feature extraction. However, their approach was limited to 2D analysis and failed to account for the complexity of 3D imaging data [24]. Similarly, Mzoughi et al [25] proposed a multiscale 3D CNN for glioma classification based on MRI, achieving approximately 85% accuracy, but still faced limitations in feature representation and generalizability.

Previous studies have systematically evaluated MRI-based radiomics and DL approaches for predicting response to nCRT in RC. A multicenter study published in *Radiology* demonstrated that MRI radiomics models could predict pathological complete response (pCR) with relatively high accuracy [26]. DL models based on post-nCRT T2W MRI also achieved discriminative performance for pCR or GR that was comparable to, or even surpassed, expert radiologist assessment [27]. Beyond single time point models, delta radiomics—which captures changes in features before and after treatment—showed superior performance in predicting nCRT response [28]. In terms of risk stratification and clinical translation, late-fusion multimodal models that integrated multiparametric MRI–derived DL or radiomics features with clinical variables outperformed unimodal models and provided greater clinical net benefit on DCA [18]. Furthermore, several systematic reviews and meta-analyses confirmed the robustness of imaging AI for predicting pCR, reporting pooled AUCs of approximately 0.90‐0.91; models based on DL or clinical fusion generally outperformed conventional radiomics approaches [29]. These findings are consistent with the results of this study and support our design rationale of introducing more advanced architectures (eg, Transformers) and evaluating clinical use using tools such as DCA.

In contrast, our Transformer-based model achieved an accuracy of 87% and an AUC of 0.921, outperforming the CNN-based approaches in both precision and discriminative capacity. The multihead self-attention mechanism of the Transformer effectively captured global dependencies within tumor regions, demonstrating enhanced robustness and generalizability, especially in cases with complex morphology or blurred tumor boundaries. Compared with previously reported models, our approach offered distinct advantages in architectural innovation, predictive performance, and clinical interpretability, positioning it as a more reliable framework for RT response prediction.

Additionally, the U-Net model also demonstrated satisfactory performance in tumor segmentation, achieving a mean DSC of 0.892 and an IoU of 0.814 on the independent test set, indicating strong consistency with expert annotations. These results underscore the crucial role of accurate segmentation in radiomics workflows, as it ensures high-quality inputs for downstream feature extraction and classification, even in tumors with irregular edges.

Furthermore, Grad-CAM visualization revealed that the Transformer model concentrated on tumor edges and regions of heterogeneous density, areas known to correlate with RT sensitivity [20,21]. This interpretability enhanced the interpretability and clinical credibility of the model.

### Limitations

Despite the promising results, this study had several limitations. First, the dataset was derived from a single center, with limited diversity in race, age, and pathological subtypes, which may have constrained the model’s generalizability [20]. Although data augmentation and k-fold cross-validation were applied to mitigate this limitation, future studies should incorporate multicenter datasets to assess the model’s robustness across broader populations. Second, the imaging data primarily consisted of DCE-MRI, without the integration of additional clinical variables such as genomic data, pathological classification, or treatment history. This single-modality approach may have limited the comprehensiveness of the predictive model [7]. Incorporating multimodal data in future models could improve both predictive accuracy and biological interpretability. Third, although Grad-CAM was used to enhance model interpretability, it functioned as a post hoc visualization method and did not directly reveal the underlying causal mechanisms driving model predictions. Future research that integrates molecular biomarkers and longitudinal follow-up data may help elucidate the biological basis of model outputs and strengthen their interpretability. Fourth, model performance evaluation was conducted on a single-center dataset and validated only on an internal holdout test set comprising 10% of the cohort. External multicenter validation was not performed. Although cross-validation and regularization techniques were used to reduce overfitting, radiomics models are known to be sensitive to cross-center variations—such as differences in imaging parameters, scanner models, and acquisition protocols—which may lead to substantial domain shift in real-world applications. Future studies should incorporate external datasets from multiple institutions to systematically assess robustness, generalizability, and clinical applicability. Fifth, expert-annotated tumor masks were used in the test set to avoid data leakage; however, this evaluation setup represents an “idealized” condition that does not fully reflect real-world clinical workflows. In practice, errors from automatic segmentation networks may propagate to the Transformer classifier, potentially leading to lower end-to-end performance than the manually masked results reported here. Future work will include validation of a fully automated pipeline and quantitative assessment of how segmentation inaccuracies affect downstream prediction tasks. Sixth, MRI slice thickness varied between 3 mm and 4 mm across patients. Although this study adopted a 2D slice–level modeling strategy, in which the inputs primarily rely on in-plane texture and morphological information, and volumetric resampling along the *z*-axis was intentionally avoided to prevent interpolation-induced artifacts, we acknowledge that differences in slice thickness and spatial resolution may still affect the statistical distributions of intensity and texture features, thereby constituting a potential confounding factor in radiomics-based analyses. We performed patient-level splitting for training, validation, and testing and used data augmentation and cross-validation to mitigate instability arising from acquisition-related heterogeneity; however, such effects cannot be completely eliminated. Future work will further explore standardized resampling strategies or explicit modeling of resolution differences (eg, stratification or covariate adjustment) and will incorporate 3D models and multicenter external validation to systematically evaluate the impact of slice thickness and scanner or protocol variability on model generalizability. Seventh, this study combined CR and PR patients into a single “GR” category to improve class balance, a strategy commonly used in statistical modeling. However, this grouping does not optimally reflect clinical practice: “watch-and-wait” strategies are typically suitable only for patients achieving clinical CR or pCR, whereas PR patients still require further treatment. While the binary classification framework in this study aimed primarily to demonstrate the DL model’s ability to predict overall RT response, future research should investigate multiclass models to enhance clinical interpretability and applicability. Eighth, although the Transformer model demonstrated superior predictive performance compared with CNN and GCN models, this improvement comes at the cost of increased computational burden. Transformers generally require larger parameter counts and higher floating-point computation, with the self-attention mechanism imposing substantial overhead when processing high-resolution image patches. To address this concern, we have supplemented the revised manuscript with a detailed comparison of model parameter counts, FLOPs, and patient-level inference time, thereby quantitatively assessing the computational efficiency of different architectures. The results demonstrate that despite its higher computational cost relative to ResNet-34 and the GCN model, the Transformer maintains a patient-level inference time within the acceptable range for real-time clinical decision support, indicating its practical feasibility for deployment under the current experimental settings. Nevertheless, in larger-scale datasets or resource-constrained clinical environments, model complexity and deployment cost may still pose limiting factors. Future work will therefore explore model compression strategies, such as knowledge distillation, structural pruning, and parameter sharing, to further reduce inference latency and computational resource consumption while preserving predictive performance, thereby enhancing the applicability of the model in real-world clinical workflows.

### Future Directions

Future research should aim to extend the application of the Transformer-based radiomics model beyond RC, exploring its transferability and adaptability to other solid tumors such as cervical, lung, and esophageal cancers, in order to evaluate cross-cancer generalizability [15,30]. Another promising avenue involves integrating radiomics with additional data modalities, including genomic, transcriptomic, immunomic, and digital pathology features, to develop comprehensive multimodal fusion models. Such models have the potential to enhance predictive accuracy and provide deeper insights into the biological mechanisms underlying treatment response [21]. Furthermore, the development of lightweight and resource-efficient model versions suitable for deployment on edge devices will be critical for facilitating real-world clinical translation, enabling the routine use of DL-based radiomics models at the point of care to support individualized treatment planning and decision-making.

### Conclusions

This study successfully constructed a Transformer-based DL radiomics model for predicting RT sensitivity in patients with RC. The results demonstrate that when using pre-RT MRI features as the primary input, the proposed model achieves superior performance in terms of predictive accuracy, interpretability, and model stability, thereby providing a feasible tool for pretreatment risk stratification and personalized RT decision-making. Supplementary analyses indicate that the multimodal integration of CT and MRI does not yield a statistically significant improvement in overall discriminative performance; however, it may offer a modest and consistent clinical net benefit at specific decision thresholds. Further validation using multicenter datasets is warranted, and future studies should more clearly delineate the potential value of multimodal information across different clinical application scenarios.

## Supplementary material

10.2196/77313 Multimedia Appendix 1 Patient inclusion flowchart for rectal cancer. The flowchart illustrates the inclusion and exclusion process. A total of 2450 patients were screened; 200 were excluded for missing imaging data, 120 for poor image quality, 80 for lack of radiotherapy outcome labels, and 50 for not receiving standard radiotherapy. Ultimately, 2000 patients were included for model analysis, with 1300 classified as radiotherapy-sensitive and 700 as radiotherapy-insensitive based on follow-up evaluations.

10.2196/77313 Multimedia Appendix 2 Scanner models and acquisition parameters used in the study.

10.2196/77313 Multimedia Appendix 3 Data collection and preprocessing flowchart. These figures illustrate the entire process from data collection, data anonymization, formatting, normalization, and data augmentation to the final dataset preparation. They depict data preprocessing steps such as denoising, cropping, normalization, and resolution adjustment.

10.2196/77313 Multimedia Appendix 4 Workflow of deep learning model training and validation. This figure illustrates the overall framework of the deep learning analysis pipeline used in this study, including data preprocessing, tumor region segmentation, feature extraction, model training with cross-validation, and performance evaluation. First, preradiotherapy images were cleaned, normalized, and augmented to generate standardized inputs. Subsequently, a U-Net model was used for automatic tumor segmentation, and the resulting masks were used to extract tumor region features from the magnetic resonance images. The Transformer model then performed classification training based on patch embeddings, leveraging multihead self-attention to capture global feature patterns. Five-fold cross-validation was adopted during training to enhance model robustness and generalizability. Finally, performance metrics, including accuracy, sensitivity, specificity, and area under the curve, were computed. This workflow provides a comprehensive overview of the deep learning analysis pathway from raw imaging data to model prediction.

10.2196/77313 Multimedia Appendix 5 U-shaped convolutional neural network (U-Net) model tumor segmentation process diagram. This diagram illustrates how the U-Net segmentation model extracts features layer by layer through convolution operations, ultimately segmenting the tumor region. It includes the original image input, convolution process, feature extraction, and segmentation results.

10.2196/77313 Multimedia Appendix 6 Schematic architecture of the transformer classification model. This figure illustrates the overall workflow of the Transformer-based classification model used to predict radiotherapy response in rectal cancer. First, pretreatment magnetic resonance images are processed using tumor-region masks (masked region of interest). The masked images are then divided into fixed-size 16×16 patches, which are linearly projected to obtain patch embeddings. The model consists of 12 Transformer encoder layers, where multihead self-attention mechanisms capture global contextual features. During classification, a dedicated classification token is passed through a multilayer perceptron (MLP head) to produce the final prediction of radiotherapy response (sensitive vs nonsensitive). This architecture operates exclusively at the image level and does not include any segmentation decoder, remaining fully independent from the U-Net segmentation model used elsewhere in the study.

10.2196/77313 Multimedia Appendix 7 Model evaluation and performance analysis flowchart. This figure illustrates the entire model evaluation process, including the calculation of accuracy, sensitivity, specificity, receiver operating characteristic curve, and area under the curve values, and how these metrics are used to assess the model's classification ability.

## Abbreviation

AI: artificial intelligence
AUC: area under the curve
CNN: convolutional neural network
CONSORT: Consolidated Standards of Reporting Trials
CR: complete response
CT: computed tomography
DCA: decision curve analysis
DCE-MRI: dynamic contrast–enhanced magnetic resonance imaging
DL: deep learning
DSC: Dice Similarity Coefficient
FLOP: Floating-Point Operation
GCN: graph convolutional network
GR: good response
Grad-CAM: Gradient-Weighted Class Activation Mapping
IoU: Intersection over Union
MRI: magnetic resonance imaging
nCRT: neoadjuvant chemoradiotherapy
pCR: pathological complete response
PR: partial response
RC: rectal cancer
RECIST: Response Evaluation Criteria in Solid Tumors
ResNets: residual networks
ROC: receiver operating characteristic
ROI: region of interest
RT: radiotherapy
T2W: T2-weighted
U-Net: U-shaped convolutional neural network
