# Supplementary figures and images for: Rectal Cancer Radiotherapy Response Prediction: Retrospective Study of Development of a Deep Learning–Based Radiomics Model
Source: JMIR Med Inform. 2026 Mar 17;14:e77313. doi: 10.2196/77313 (PMC12994884; doi:10.2196/77313)

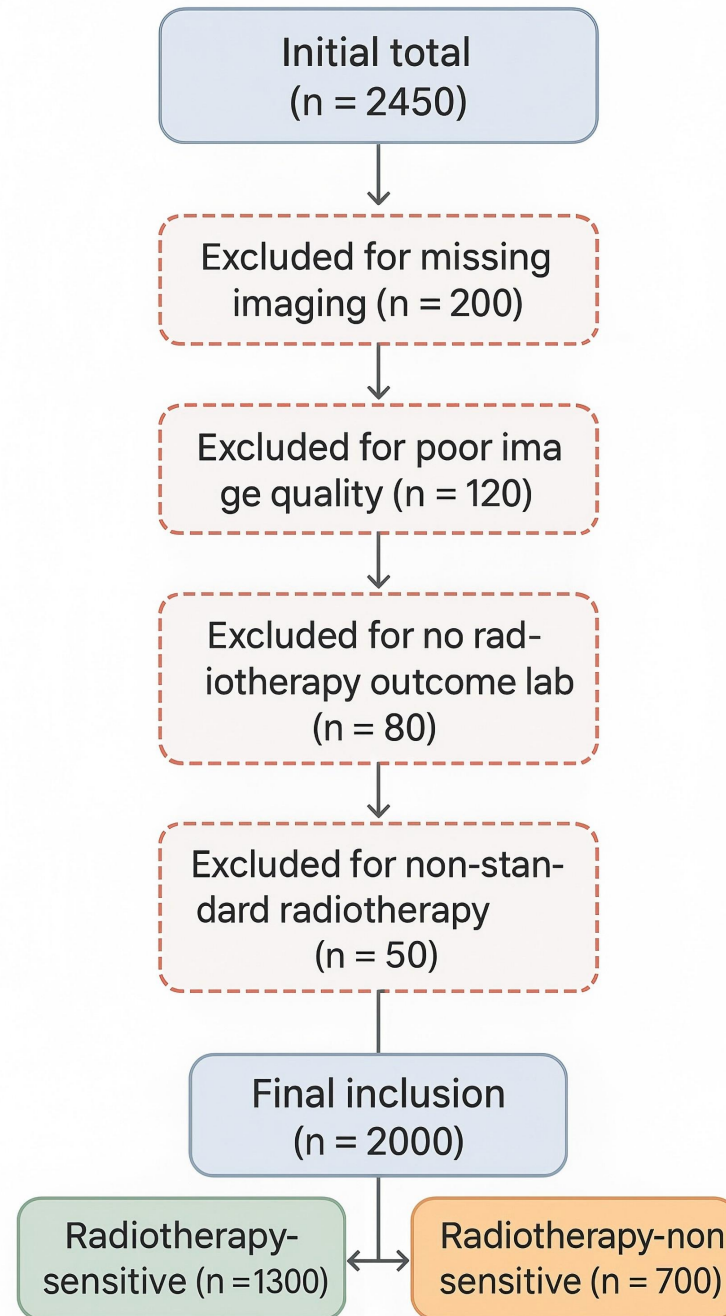

Supplement: Multimedia Appendix 1 [file medinform-v14-e77313-s001.pdf]

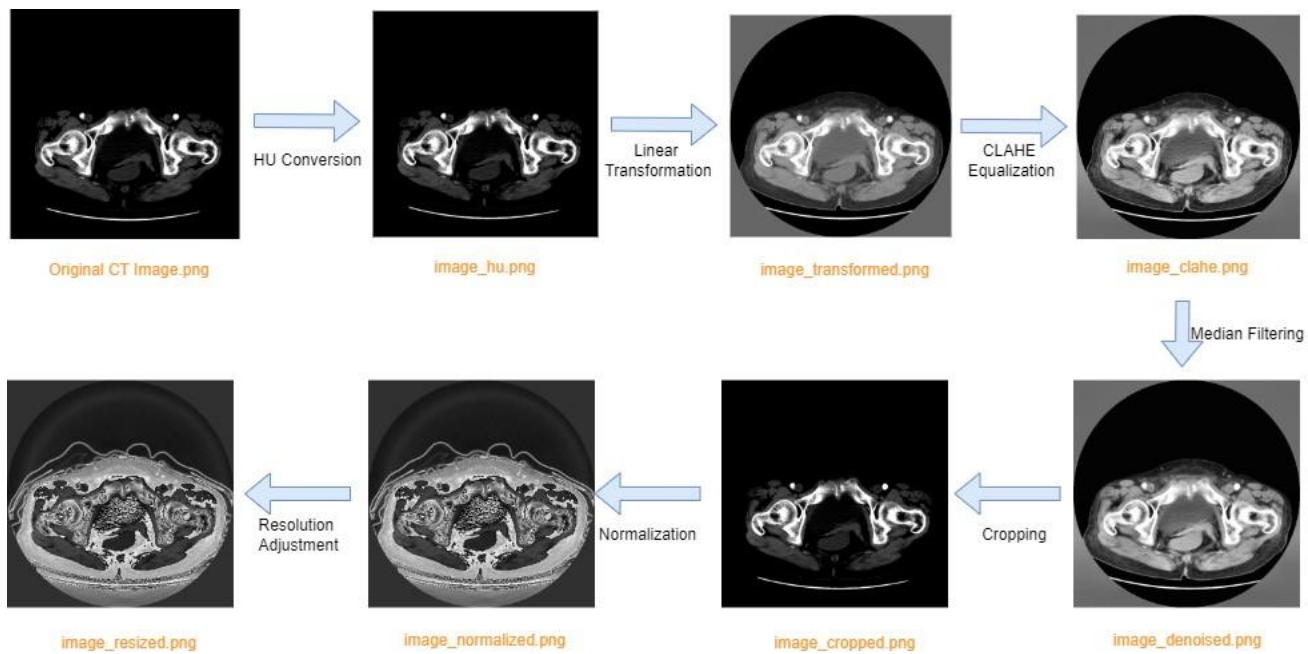

Supplement: Multimedia Appendix 3 [file medinform-v14-e77313-s003.pdf]

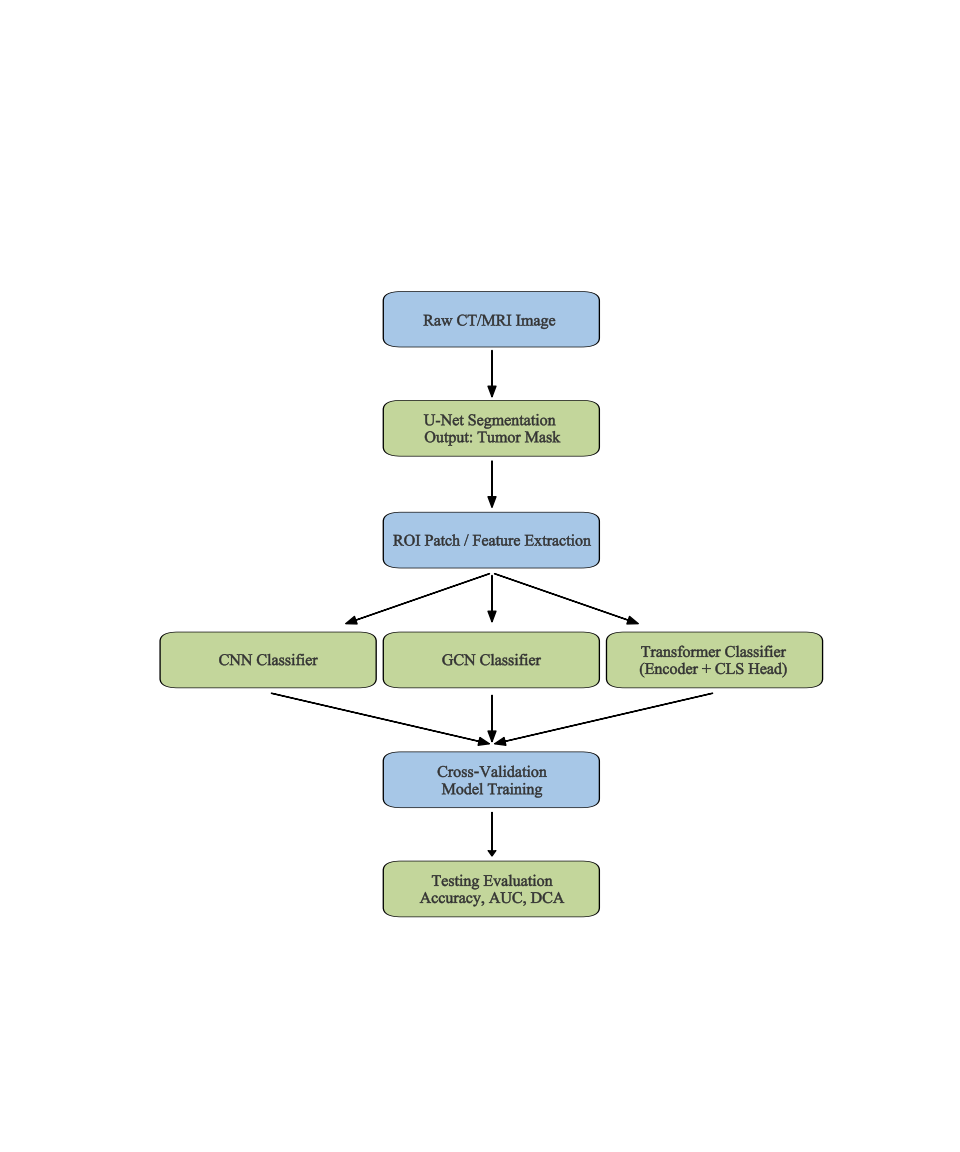

Supplement: Multimedia Appendix 4 [file medinform-v14-e77313-s004.png]

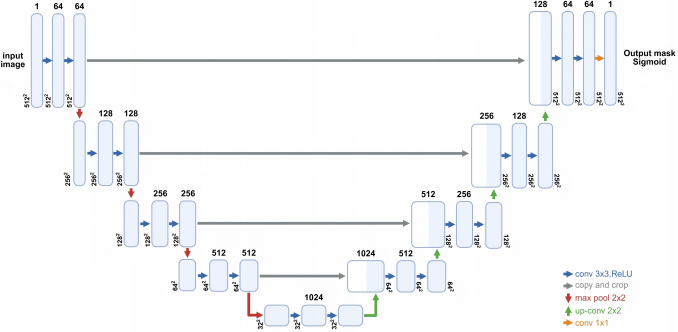

Supplement: Multimedia Appendix 5 [file medinform-v14-e77313-s005.png]

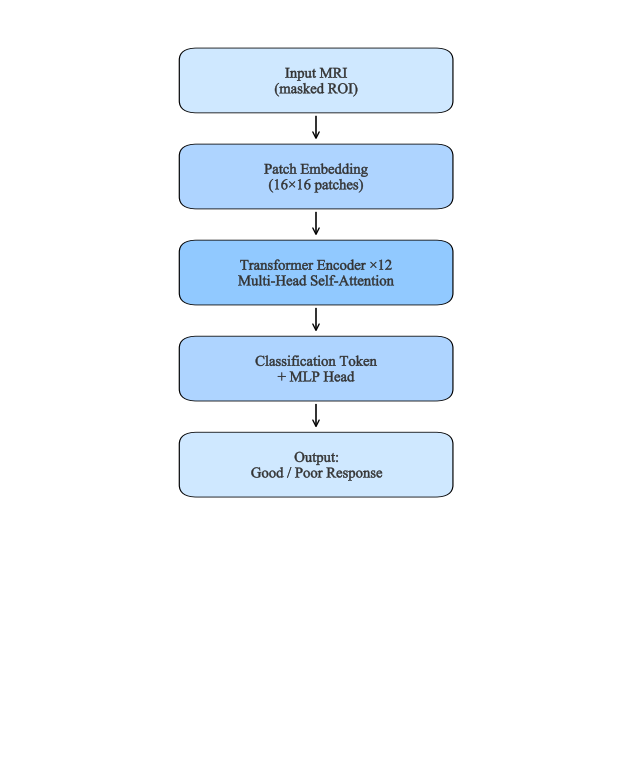

Supplement: Multimedia Appendix 6 [file medinform-v14-e77313-s006.png]

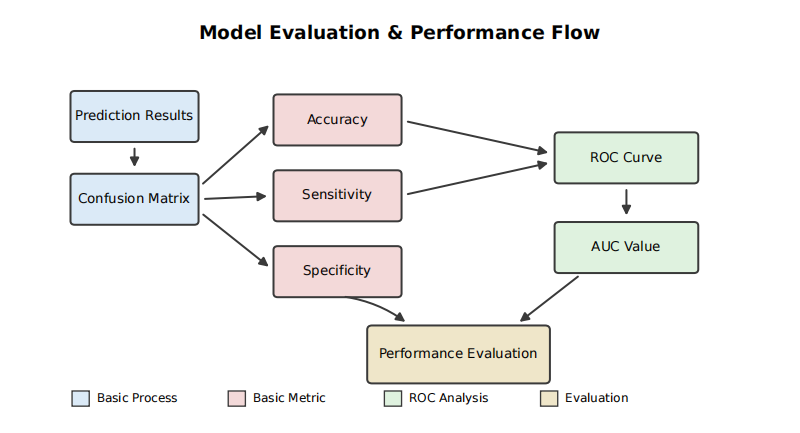

Supplement: Multimedia Appendix 7 [file medinform-v14-e77313-s007.png]
