# Supplementary material for: Rectal Cancer Radiotherapy Response Prediction: Retrospective Study of Development of a Deep Learning–Based Radiomics Model
Source: JMIR Med Inform. 2026 Mar 17;14:e77313. doi: 10.2196/77313 (PMC12994884; doi:10.2196/77313)
Supplement: Multimedia Appendix 2 [file medinform-v14-e77313-s002.docx]

| **Table S1. Scanner models and acquisition parameters used in the study** | | | | | |
| --- | --- | --- | --- | --- | --- |
| Modality | Manufacturer | Model | Field strength / Slice thickness | Acquisition range (year) | No. of patients (%) |
| MRI | Siemens | MAGNETOM Aera | 1.5 T / 4 mm | 2015–2018 | 620 (31.0%) |
| MRI | GE | Discovery MR750 | 3.0 T / 3–4 mm | 2018–2023 | 1,050 (52.5%) |
| MRI | Philips | Ingenia CX | 3.0 T / 3 mm | 2020–2023 | 330 (16.5%) |
| CT | Siemens | SOMATOM Definition AS+ | 120 kVp / 2.5 mm | 2015–2023 | 1,200 (60.0%) |
| CT | GE | Revolution EVO | 120 kVp / 2.5 mm | 2019–2023 | 800 (40.0%) |

**Note:** All scanners underwent standard quality assurance procedures. Image preprocessing steps (normalization and resampling) were applied to mitigate inter-scanner variability prior to model training.
